# Prothesis and Re-education of the Mutilés

**Published:** 1919-01

**Authors:** 


**Prothesis and Re-education of the Mutilés.** By Prof. A. Broca. *Revue Interalliée pour les Mutilés de la Guerre.* April, 1918.

Artificial limbs for mutilés theoretically should restore as well as possible both the external appearances and functions, but Prof. Broca fears that in many cases the esthetic point of view is taken into consideration more than the practical one.

For a man with an amputation of the arm who wishes to do manual work, an artificial hand in the form of a hand is of no use at all. He needs much more the very simplest artificial arm or fore-arm, supplied with a hook, a ring, or tongs. These tools can be made useful; they are articulated and may be interchanged for the different cases.

A man with an amputation of the right hand should, first of all, learn to use his left hand as much as possible. His right needs only to be replaced by a passive tool, serving to hold the objects while the left hand does the work.

Quite a number of " working-hands " have been devised and realized. They should be as light, solid and practical as possible, and should be interchangeable and well adapted to certain trades or even to certain definite processes, for generally a disabled workman needs many of them successively.

